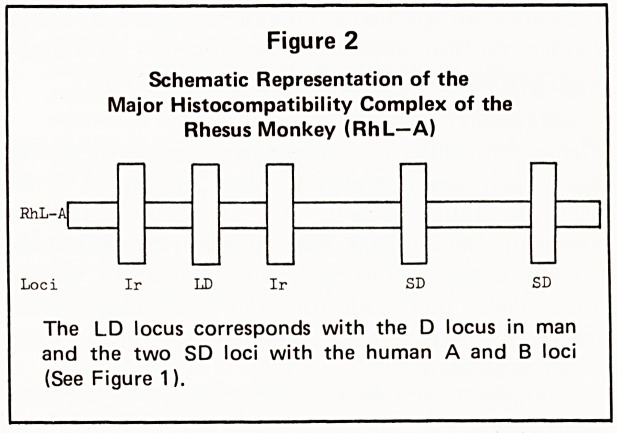# Associations between HLA Antigens and Disease
*Based on a lecture given in a Symposium 'Cellular Antigens and Disease' at the 30th Annual Meeting of the American Association of Blood Banks, Atlanta, USA, 15th November 1977.


**Published:** 1978

**Authors:** Geoffrey H. Tovey


### Associations between HLA Antigens and Disease*

Geoffrey H. Tovey, C.B.E., M.D., F.R.C.P., F.R.C.Path.†

Tissue matching for human leucocyte antigens (HLA) is important for graft survival in transplantation of organs such as skin, kidney or cornea. More surprisingly, however, a number of human diseases occur more frequently in persons who have inherited particular HLA antigens. Genes controlling the inheritance of the HLA antigens are located on chromosome 6, and the major histocompatibility complex (MHC) in man involves at least four gene loci (A, B, C and D) controlling at least 64 HLA specificities. Recently, certain genes have been identified which code for a group of HLA alloantigens demonstrable on B-cell lymphocytes, but not on T-lymphocytes or platelets.[1] In the mouse, B-lymphocyte antigens are referred to as IA, or immune response-associated antigens and, initially therefore, human B-cell antigens were referred to as wIA. Some, if not all, such antigens are, however, closely related to the products of the HLA-D locus[2] and the WHO Tissue Typing Nomenclature Committee has currently designated them DR (for D related), followed by the letter 'w' to indicate that their designation is still provisional. The probable gene structure of the MHC on chromosome 6 is outlined in Figure 1.

The extensive search for associations between HLA antigens and specific diseases in man was stimulated by the demonstration of genetic linkage in mice between the murine histocompatibility complex, H—2, and resistance to virus induced leukaemias. Lilly and his colleagues[3] found that strains of mice can be bred which show susceptibility or resistance to leukaemia, and that in certain strains the disease will progress rapidly. Leukaemic mice carrying the histocompatibility antigen $H{-}2^b$, for example, survive longer than those carrying $H{-}2^k$. A number of human diseases, some of which are known or suspected to result from disordered immune processes, are now considered to be associated with particular HLA—A, B and D antigens.[4] More recently, an even closer association between certain DR (B-lymphocyte) alloantigens and some of these diseases has been identified.[5]

The strength of an association is expressed as the relative risk, which indicates how many times more frequently the disease is found in individuals positive for a particular antigen relative to those negative for that determinant. Many of the postulated associations are somewhat tenuous and I propose in this review, therefore, to list only those diseases which are found at least four times more often in persons possessing the specific HLA antigen. These are shown in Tables 1 and 2 and are combined estimates for the reported studies.

**Figure 1**

**Schematic Representation of the Major Histocompatibility Complex in Man (HLA)**

The DR locus, whose determinants are preferentially expressed on B cells, lies in close proximity to the D or MLC locus. Although the D and DR alloantigens may be products of the same locus, they are not necessarily identical.

### DISEASES ASSOCIATED WITH HLA ANTIGENS OF THE A SERIES

Until recently, no association had been reported of a four-fold relative risk between a disease and an antigen of the A series. Myasthenia gravis and coeliac disease show some association with HLA—A1 (relative risks 2.5 and 2.7 respectively), whilst

*Based on a lecture given in a Symposium 'Cellular Antigens and Disease' at the 30th Annual Meeting of the American Association of Blood Banks, Atlanta, USA, 15th November 1977.

†Consultant Adviser in Blood Transfusion, DHSS. Late Director, SW Blood Transfusion Service, Southmead.

**Table 1**

**HLA—B Antigens and Disease Association**

| Antigen | Disease | Incidence of Antigen (%) Patients | Controls | Average Relative Risk* |
|---|---|---|---|---|
| B7 | Multiple sclerosis | 56 | 20 | 5 |
| | Ragweed hay fever | 50 | 20 | 5 |
| B8 | Coeliac disease | 67—87 | 24 | 15 |
| | Dermatitis herpetiformis | 60—90 | 24 | 15 |
| | Juvenile dermatomyositis | 75 | 21 | 11 |
| | Addison's disease | 80 | 24 | 8 |
| | Idiopathic thrombocytopenic purpura | 70 | 20 | 8 |
| | Chronic active hepatitis | 62 | 20 | 5 |
| | Sjogren's syndrome | 58 | 21 | 5 |
| | Myasthenia gravis | 59 | 20 | 4 |
| B13 | Psoriasis vulgaris | 27 | 3 | 4 |
| B14 | Idiopathic haemochromatosis | 20—25 | 4 | 8 |
| | Leprosy | 23 | 4 | 7 |
| B15 | Systemic lupus erythematosus | 46 | 8 | 7 |
| B18 | Multiple myeloma | 35 | 9 | 5 |
| | Hodgkin's lymphadenoma | 29 | 9 | 4 |
| B27 | Ankylosing spondylitis | 90—96 | 6 | 96 |
| | Reiter's syndrome | 68—81 | 6 | 45 |
| | Anterior uveitis | 55—71 | 6 | 15 |
| | Juvenile rheumatoid arthritis | 29 | 6 | 4 |
| Bw35 | Sub-acute thyroiditis | 86 | 19 | 6 |
| | Thyrotoxicosis | 57 | 20 | 5 |

*The relative risk (X) for a particular determinant is calculated by the the formula: $X = \frac{ad}{bc}$ where: a = the number of positive patients; b = the number of negative patients; c = the number of positive controls and d = the number of negative controls.

multiple sclerosis has an even more tenuous association with HLA—A3, with a relative risk of 1.7. In May 1976, however, Simon et al[6] reported a significant association between HLA—A3 and idiopathic haemochromatosis, A3 being present in 78 per cent of 51 affected patients, compared with a frequency of 27 per cent in normal controls, and giving a relative risk of 8.2. Bomford and his colleagues[7] have confirmed this association, finding HLA—A3 to be present in 69 per cent of a further 35 unrelated patients.

### HLA—B ANTIGENS

Although almost as many HLA—A antigens have been identified as HLA—B, most of the disease associations found so far in man are with alleles of the B locus and are summarised in Table 1. Because of the postulated close proximity of the B and D loci on chromosome 6, it was to be expected that some of these diseases would also show an association with the HLA—D antigens, and this has, in fact, proved to be the case (see below).

### HLA—D ANTIGENS

HLA antigens of the A and B series are detected by serological methods (the microlymphocytotoxicity test), but antigens of the D series can only be detected by one-way mixed lymphocyte culture (MLC). This more complicated technique has not been widely used and fewer studies have therefore been made for HLA—D and disease associations. The most significant of these are given in Table 2.

HLA—Dw3 is often found in close linkage with HLA—B8. Not surprisingly, therefore, most of the diseases found to be significantly associated with HLA—B8 are associated also with HLA—Dw3.

In patients with multiple sclerosis, not only was the frequency of HLA—Dw2 increased (70% compared with 16% in healthy controls), but the clinical progression of the disease was significantly more rapid in those who were Dw2 positive.[8]

### HLA—DR ANTIGENS

Studies of the clinical relevance of the DR (B—lymphocyte) antigens are only very recent, but already a number of independent investigations has indicated an association between HLA—DRw2 and multiple sclerosis that is possibly stronger than between MS and the A, B and D locus antigens.[9, 10] In a survey instigated by the 7th International Histocompatibility Workshop (1977), however, in which strict clinical criteria for MS were proscribed, although the association with DRw2 was confirmed, it was not as strong as that between MS and B7 or Dw2. This same Workshop reported also an association between DRw3 and chronic active hepatitis, and in this study the relative risk for this disease and the antigens DRw3, Dw3 and B8 was essentially similar (i.e. approximately five-fold).

Schernthaner, Ludwig and Mayr[11] found that individuals positive for DRw3 carry a four-fold

**Table 2**

**HLA—D Antigens and Disease Associations**

| Antigen | Disease | Relative Risk |
|---|---|---|
| Dw2 | Multiple sclerosis | 5 |
| Dw3 | Coeliac disease | 16 |
| | Addison's disease | 10 |
| | Sjogren's disease | 8 |
| | Thyrotoxicosis | 6 |
| | Chronic active hepatitis | 5 |
| | Diabetes mellitus | 4 |

increased risk of developing insulin-dependent diabetes, and Solheim et al.,[12] claim an association between DRw3 and dermatitis herpetiformis as striking as that between ankylosing spondylitis and HLA—B27, with a relative risk of 84. This latter finding is so exceptional that it will be interesting and important to see whether it is confirmed in further independent studies.

### DISCUSSION

Various hypotheses have been proposed to explain the associations between HLA determinants and disease.[4, 13] Little is known of the mechanisms by which susceptibility is conferred: it is unlikely, however, that the association is to the HLA genes themselves but rather to closely linked genes which may accompany them.

### INVOLVEMENT WITH IMMUNE RESPONSE (Ir) GENES

Because most disease associations are with antigens of the B and D series and many of the diseases associated with HLA—B8 and Dw3 are autoimmune disorders, it has been postulated that there are loci on chromosome 6 for immune response genes in close proximity to HLA—B and D, and that antigens of the B and D series act as 'markers' for such Ir genes. Supportive evidence that this may be so has been provided by Balner et al.[14] who have demonstrated in rhesus monkeys an Ir gene locus on either side of an MLC (or D) locus, plus loci for two series of serologically determined antigens comparable to the A and B series antigens in man (Figure 2). The recently established association of DRw2 with multiple sclerosis and DRw3 with chronic hepatitis, together with the inter-relationship of the DR and D antigens, adds further support to an involvement with Ir genes. There is evidence that DR determinants are intimately involved in the handling of antigenic material by macrophages as well as in many aspects of the regulatory effects of T cells on B cells,[15] so that an explanation favoured by many is that Ir genes in the HLA region result in the individual either failing to produce immunity against disease-causing agents or over-reacting. Attention has already been drawn to the more rapid clinical progression of multiple sclerosis in patients who are Dw2 positive and, in a study of myasthenia gravis, Feltkamp et al.[16] found that antibodies to skeletal muscle were much rarer in those who were HLA—B8 positive and that such patients acquired the disease at an earlier age. By

**Figure 2**

**Schematic Representation of the Major Histocompatibility Complex of the Rhesus Monkey (RhL—A)**

The LD locus corresponds with the D locus in man and the two SD loci with the human A and B loci (See Figure 1).

contrast, HLA—A2 positive patients more often had thymomas and antibodies to skeletal muscle but tended to develop myasthenia gravis at an older age.

An association between B8 and chronic active hepatitis has been reported and there is an inverse correlation between HLA—B8 and the persistence of hepatitis B surface antigen ($HB_sAg$) in the patient's serum.[17] Galbraith and his colleagues[18] suggest that the allele for B8 is linked to genes that promote abnormally raised and prolonged antibody responses, so that persons who are HLA—B8 positive rapidly become $HB_sAg$ negative but go on to develop an aggressive chronic hepatitis and more severe liver damage. Bailey et al.[19] found cirrhosis more likely to occur in persons with alcoholic liver disease if they are B8 positive, and that raised serum IgA and IgG concentrations are more common in those patients who progress to cirrhosis. There is also an association in diabetics between B8 and the development of pancreatic islet-cell antibodies. In a study of insulin-dependent diabetics, Morris et al.[20] found that 61 per cent of those with antibodies were B8 positive by comparison with only 35 per cent in diabetics without islet-cell antibodies. This increased frequency of B8 was even higher (71%) in those in whom antibodies had persisted for more than 5 years.

### MOLECULAR MIMICRY AND RECEPTOR MECHANISM

The strongest association yet discovered between an HLA antigen and a disease is between HLA—B27 and ankylosing spondylitis, and this has been confirmed in other races.[21] It has been suggested therefore that a given tissue antigen may so closely resemble a particular virus that carriers of the allele fail to recognise the virus as 'non-self' and thus have a poor immunological defence against it. An alternative proposal is that some HLA determinants may act as receptors for particular infectious agents and thus render the host unusually susceptible to invasion by such pathogens.[4]

COMPLEMENT FACTORS

Genes controlling the complement factors C2 and C4 have been demonstrated in close proximity to the MHC on chromosome 6.[22, 23] It has been proposed therefore that linkage disequilibrium between some HLA and the complement genes may exist, so that some alleles then code for defective products which interfere with the elimination of various micro-organisms.

None of these hypotheses is entirely satisfactory. Even in the situation in which there is an association as striking as that between HLA—B27 and ankylosing spondylitis, fewer than 5 per cent of individuals positive for this antigen will develop the disease and, even within affected families, the disease does not show 100 per cent linkage with the B27 antigen.[24] Such family studies, and others in cases of asthma and idiopathic haemochromatosis, suggest that disease susceptibility genes at more than one locus on chromosome 6 may be involved and that these may be affected by a 'penetrance' gene, or genes, showing linkage disequilibrium with HLA. Possibly the mechanism or the association is different in various diseases and not one but several of the above hypotheses will turn out to be true.

Apart from ankylosing spondylitis, and perhaps dermatitis herpetiformis, HLA typing a patient is of little value in the diagnosis of disease. Since, however, ankylosing spondylitis may sometimes present with vague and indefinite symptoms, checking whether the patient is positive for HLA—B27 may be of help to the rheumatologist in the doubtful case. Probably the more important clinical application of these studies is the potential they offer for the sub-division, and more effective clinical management, of some well established diseases. Irvine et al.[25] claim that those who develop so-called 'maturity-onset' diabetes and are HLA—B8 positive are more likely to require insulin than those who are B8 negative. In a similar study, Irvine, Gray, Morris and Ting[26] have found that thyrotoxic patients who are B8—positive are nearly twice as likely to relapse after the withdrawal of antithyroid drugs than those who are B8 negative. Sjogren's syndrome is only sometimes accompanied by arthritis, and Chused et al.[27] report an association with this syndrome and both B8 and Dw3, but only in those patients without arthritis. Finally, Ungar et al.[28] in a study of 127 patients with pernicious anaemia found that when PA was associated with multiple endocrine disorders there was an increased incidence of B8, by contrast with those patients with PA but no endocrine disease in whom there was an increase in HLA—B7 and HLA—B12.

In summary, therefore, it would seem likely that in man, as in the mouse and the rhesus monkey, we have as a part of our major histocompatibility complex, not only genes which determine how our tissues will react to transplantation antigens in skin, kidneys, corneae and other organs, but also those which control our response to the introduction of foreign antigens in the form of viruses and bacteria. These may be regarded as 'immune response' or 'disease susceptibility' genes, and are probably clustered around the HLA—B, D and DR loci on chromosome 6. It seems likely that they are remote from the HLA—A and C gene loci, since only one disease has been found significantly associated with a product of either of these loci (i.e. HLA—A3 and idiopathic haemochromatosis). As yet, no four-fold association between an HLA—C antigen and a particular disease has been demonstrated, although a recent report claims that those who possess the antigen Cw6 are three times more likely to develop psoriasis than those who are Cw6 negative.[29]

### REFERENCES

1. MICKEY, M. R. (1975). In *Histocompatibility Testing 1975*, (Ed.) F.Kissmeyer-Nielsen, Munksgaard, Copenhagen, pp. 657—59.
2. BODMER, J., PICKBOURNE, P., BODMER, W., BATCHELOR, R. J., DEWAR, P., DICK, H. and ENTWISTLE, C. (1976). Serological identification of Ia antigens: report of a British Region Ia Workshop. *Tissue Antigens,* **8,** 359—372.
3. LILLY, F., BOYSE, E. A. and OLD, L. J. (1964). Genetic basis of susceptibility to viral leukemogenisis. *Lancet,* **2,** 1207—1209.
4. SVEJGAARD, A., PLATZ, P. and RYDER, L. P. (1975). HLA and disease associations. *Transplantation Reviews,* **22,** 3—43.
5. MANN, D. L., KATZ, S. I., NELSON, D. L., ABELSON, L. D. and STRUBER, W. (1976). Specific B-cell antigens associated with gluten-sensitive enteropathy and dermatitis herpetiformis. *Lancet,* **1,** 110—111.
6. SIMON, M., BOUREL, M., FOUCHET, R. and GENETET, B. (1976). Association of HLA—A3 and HLA—B14 antigens with idopathic haemochromatosis. *Gut,* **17,** 332—334.
7. BOMFORD, A., EDDLESTON, A. L. W. F.,

KENNEDY, L. A., BATCHELOR, J. R. and WILLIAMS, R. (1977). Histocompatibility antigens as markers of abnormal iron metabolism in patients with idopathic haemochromatosis and their relatives. *Lancet,* **1**, 327–329.

8. JERSILD, C., FOG, T., HANSEN, G. S., THOMSEN, M., SVEJGAARD, A. and DUPONT, B. O. (1973). Histocompatibility determinants in multiple sclerosis, with special reference to clinical course. *Lancet,* **2**, 1221–1225.
9. COMPSTON, D. A. S., BATCHELOR, R. J. and McDONALD, W. I. (1976). B-lymphocyte alloantigens associated with multiple sclerosis. *Lancet,* **2**, 1261–1265.
10. TERASAKI, P. I., PARK, M. S., OPELZ, G. and TING, A. (1976). Multiple sclerosis and high incidence of a B-lymphocyte antigen. *Science,* **193**, 1245–1247.
11. SCHERNTHANER, G., LUDWIG, H. and MAYR, W. R. (1977). B-lymphocyte alloantigens and insulin-dependent diabetes mellitus. *Lancet,* **2**, 1128.
12. SOLHEIM, B. G., ALBRECHTSEN, D., THORSBY, E. and THUNE, P. (1977). Strong association between an HLA–Dw3 associated B cell alloantigen and dermatitis herpetiformis. *Tissue Antigens,* **10**, 114–118.
13. McDEVITT, H. O. and BODMER, W. F. (1974). HLA, immune-response genes, and disease. *Lancet,* **1**, 1269–1275.
14. BALNER, H., VAN VREESWIJK, W. and ROGER, J. H. (1976). Ia-like antigens of rhesus monkeys. *Transplantation Reviews,* **30**, 3–17.
15. TAUSSIG, M. J. and MUNRO, A. J. (1974). *Nature* (London), **251**, 63–65.
16. FELTKAMP, T. E. W., van den BERG-LOONEN, P. M., NIJENHUIS, L. E., ENGELFRIET, C. P., van LOGHEM, J. J., van ROSSUM, A. L. and OOSTERHUIS, G. H. (1974). Myasthenia gravis, autoantibodies and HLA antigens. *British Medical Journal,* **1**, 131–133.
17. LINDBERG, J., IWARSON, S. and LINDHOLM, Annika. (1977). Genetic factors in the development of chronic active hepatitis. *Lancet,* **1**, 67–68.
18. GALBRAITH, R. M., WILLIAMS, R., PATTISON, J., KENNEDY, L. A., EDDLESTON, A. L. W. F., WEBSTER, A. D. B., DONIACH, D. and BATCHELOR, R. J. (1976). Enhanced antibody responses in active chronic hepatitis. *Lancet,* **1**, 930–934.
19. BAILEY, R. J., KRASNER, N., EDDLESTON, A. L., WILLIAMS, R., TEE, D. E. H., DONIACH, D., KENNEDY, L. A. and BATCHELOR, R. J. (1976). Histocompatibility antigens, autoantibodies and immunoglobulins in alcoholic liver disease. *British Medical Journal,* **2**, 727–729.
20. MORRIS, P. J., IRVINE, W. J., GRAY, R. S., DUNCAN, L. J. P., VAUGHAN, H., McCALLUM, F. J., CAMPBELL, C. J. and FARQUHAR, J. W. (1976). HLA and pancreatic islet cell antibodies in diabetes. *Lancet,* **2**, 652–653.
21. SONOZAKI, H., SEKI, H., CHANG, S., OKUYAMA, M. and JUJI, T. (1976). Human lymphocyte antigen HLA–B27 in Japanese patients with ankylosing spondylitis. *Tissue Antigens,* **5**, 131–136.
22. FU, S. M., JUNTEL, H. G. and BRUSMAN, H. P. (1974). Evidence for linkage between HLA histocompatibility genes and those involved in the synthesis of the second component of complement. *Journal of Experimental Medicine,* **140**, 1108–1111.
23. JERSILD, C., RUBINSTEIN, P. and DAY, N. K. (1976). The HLA system and inherited deficiencies of the complement system. *Transplantation Reviews,* **32**, 43–71.
24. DICK, HEATHER M., DICK, W. C., STURROCK, R. D. and BUCHANAN, W. W. (1974). Inheritance of ankylosing spondylitis and HLA antigen W27. *Lancet,* **2**, 24–25.
25. IRVINE, W. J., GRAY, R. S., McCALLUM, C. J. and DUNCAN, L. J. P. (1977). Clinical and pathogenic significance of pancreatic-islet-cell antibodies in diabetics. *Lancet,* **1**, 1025–1027.
26. IRVINE, W. J., GRAY, R. S., MORRIS, P. J. and TING, A. (1977). Correlation of HLA and thyroid antibodies with clinical course of thyrotoxicosis treated with antithyroid drugs. *Lancet,* **2**, 898–900.
27. CHUSED, T. M., KASSAN, S. S., OPELZ, G., MOUTSOPOULOS, H. M. and TERASAKI, P. I. (1977). Sjogren's syndrome associated with HLA–Dw3. *New England Journal of Medicine,* **296**, 895–897.
28. UNGAR, B., MATHEWS, J. D., TAIT, B. D. and DOWLING, D. C. (1977). HLA patterns in pernicious anaemia. *British Medical Journal,* **1**, 798–800.
29. ***HISTOCOMPATIBILITY TESTING 1977.*** (Eds.) E. F. Bodmer, J. R. Batchelor, et al., Munksgaard, Copenhagen.

## Figures and Tables

**Figure 1 f1:**
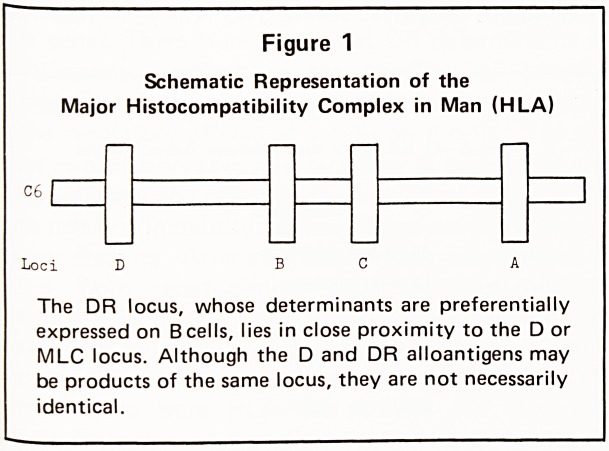


**Figure 2 f2:**